# Depicting HIV-1 Transcriptional Mechanisms: A Summary of What We Know

**DOI:** 10.3390/v12121385

**Published:** 2020-12-03

**Authors:** Antoine Dutilleul, Anthony Rodari, Carine Van Lint

**Affiliations:** Service of Molecular Virology, Department of Molecular Virology (DBM), Université Libre de Bruxelles (ULB), 6041 Gosselies, Belgium; Antoine.Dutilleul@ulb.be (A.D.); arodari@ulb.ac.be (A.R.)

**Keywords:** HIV-1, HIV-1 transcriptional regulation, HIV-1 latency

## Abstract

Despite the introduction of combinatory antiretroviral therapy (cART), HIV-1 infection cannot be cured and is still one of the major health issues worldwide. Indeed, as soon as cART is interrupted, a rapid rebound of viremia is observed. The establishment of viral latency and the persistence of the virus in cellular reservoirs constitute the main barrier to HIV eradication. For this reason, new therapeutic approaches have emerged to purge or restrain the HIV-1 reservoirs in order to cure infected patients. However, the viral latency is a multifactorial process that depends on various cellular mechanisms. Since these new therapies mainly target viral transcription, their development requires a detailed and precise understanding of the regulatory mechanism underlying HIV-1 transcription. In this review, we discuss the complex molecular transcriptional network regulating HIV-1 gene expression by focusing on the involvement of host cell factors that could be used as potential drug targets to design new therapeutic strategies and, to a larger extent, to reach an HIV-1 functional cure.

## 1. Introduction

Over the past decades, the introduction of the combinatory antiretroviral therapy (cART) has drastically reduced globally AIDS-related morbidities and increased the life span of HIV-1 infected patients. However, cART does not completely eradicate HIV-1 from the body of treated individuals and thus has to be taken throughout the patients’ lives. Therefore, the use of cART has switched the HIV-1 infection from a lethal disease to a chronic one [1,2,3]. Indeed, cART fails to reach an HIV-1 functional cure due to the existence of viral latent reservoirs corresponding to specific cell-type or anatomical tissues where the viral replication is stable [3]. The molecular mechanisms of viral latency mainly occur at the transcriptional level including transcriptional interference, chromatin organization, absence of cellular host factors, and epigenetic modifications. Overall, due to these viral latency mechanisms, the viral reservoirs are less sensitive and/or accessible to cART and are therefore persisting during the treatment, making them the main barrier to eradicate HIV infection [4,5]. Indeed, as soon as the cART treatment is interrupted, a rapid viral rebound from those latent reservoirs is observed, therefore leading to a relapse of the disease. To solve this issue, new therapeutic approaches such as the “shock and kill” and “block and lock” strategies have been developed in order to either reactivate or definitively block HIV-1 transcription in the latent reservoirs, respectively. While the “shock and kill” therapy, applied in combination with cART, aims to reach the goal of an HIV-1 functional cure, the “block and lock” strategy aims to free HIV-1 infected patients from cART treatment, since its main objective is to allow the immune system to handle the infection by suppressing viral reactivation (reviewed in [6,7]). Because these therapies are mainly targeting HIV-1 transcription, their development requires a detailed understanding of the *cis*- and *trans*-regulatory viral transcription mechanisms. In this review, we will discuss the complex network regulating HIV-1 gene expression by focusing on the role of host cell factors that may serve as potential drug targets for the development of therapeutic strategies.

## 2. Regulation of HIV-1 Basal Transcription

**Figure 1:**
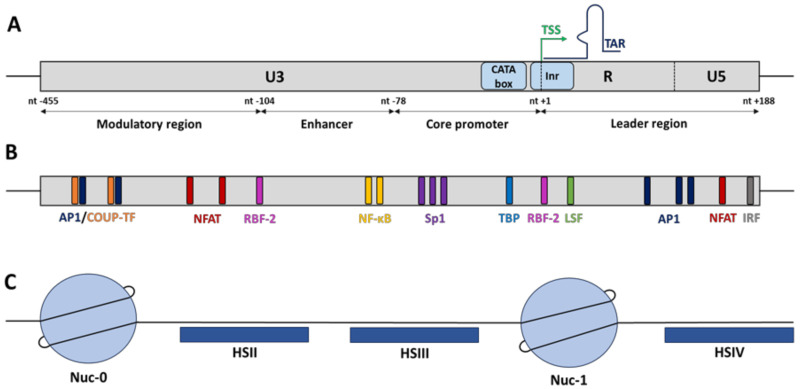
Schematic representation of the HIV-1 5′-long terminal repeat (5′LTR) promoter. (**A**) The 5′LTR can be divided into four functional domains: the modulatory region (from nt −455 to nt −104), the enhancer (from nt −109 to nt −79), the core promoter (from nt −78 to nt −1), and the leader region (from nt +1 to nt +188). (**B**) These functional domains contain numerous *cis*-regulatory elements specific to various host cellular factors. The transcriptional start site (TSS) is located at the junction between the U3 and R regions. (**C**) The HIV-1 5′LTR harbours a specific nucleosomal chromatin organization. Under latency conditions, two major DNaseI hypersensitive sites (HS), named HSII (from nt −234 to nt −132) and HSIII (from nt −67 to nt −7), are framed by two nucleosomes, nuc-0 and nuc-1. The nucleosome nuc-1 is located downstream of the TSS and plays a critical role in HIV-1 transcription regulation.

Once randomly integrated into the host cellular genome, HIV-1 gene expression is mainly regulated at the transcriptional level by hijacking the cellular RNA polymerase II (RNAPII) machinery. HIV-1 transcription initiates at the U3/R junction in the 5′-long terminal repeat (5′LTR) and is regulated by cellular transcription factors for which many binding sites have been identified in the 5′LTR and by the chromatin status of the HIV provirus established through epigenetic modifications (Figure 1) [8,9].

### 2.1. The cis-Regulatory HIV-1 5′LTR Promoter

In basal conditions, the HIV-1 core promoter activity is positively or negatively regulated independently of the viral transactivator Tat by cellular host factors that recognized multiple *cis*-regulatory elements along the 5′LTR but also by its epigenetic profile, through the recruitment of chromatin-modifying enzymes [9]. Functionally, the HIV-1 5′LTR is subdivided into four domains (Figure 1A): the modulatory region (from nucleotide (nt) −455 to nt −104 where the nt +1 is defined as the first nucleotide of the transcription start site), the enhancer (from nt −109 to nt −79), the core promoter (from nt −78 to nt −1), and the leader region (from nt +1 to nt +188) [10,11]. Overall, all these functional regions contain numerous *cis*-regulatory elements that are recognized by both constitutive transcription factors such as Sp1 (specificity protein 1) and Oct-1 (o-binding protein 1) and inducible transcription factors such as NF-κB (nuclear factor-kappa B), AP-1 (activator protein 1), and NFAT (nuclear factor of activated T-cells) that are able to enhance or repress HIV-1 transcription (reviewed in [11,12]) (Figure 1B). The outcome of viral transcriptional activity is dependent on the balance between activators and/or repressors binding to the 5′LTR. Among these transcription factors, only a few have been described as key elements to initiate HIV-1 transcription (including NF-κB, Sp1 and the TATA-box binding protein (TBP)), while others (including NFAT, C/EBP, Est-1, and AP-1) modulate the promoter activity without being essential for viral transcription [8,13,14].

#### 2.1.1. NF-kB

The 5′LTR enhancer region has been shown to strongly increase HIV-1 promoter activity but also heterologous promoter activities. Located just upstream of the HIV-1 core promoter, the enhancer region contains one of the strongest *cis*-regulatory elements, corresponding to multiple binding sites for NF-κB, an inducible factor known to be important for HIV-1 transcriptional activation [10]. While most HIV-1 subtypes have two NF-κB binding sites, the subtype C contains three to four NF-κB binding sites, associated with a putative increase in viral fitness, therefore highlighting the importance of NF-κB factors as powerful activators of HIV-1 transcription [15]. Under unstimulated or latent condition, the heterodimer p50/p65 is sequestered into the cytoplasm due to its interaction with the inhibitory protein IκBα, hiding its nuclear localization signal. However upon stimulation, typically following PKC activation or through the MAPK signalling pathway, the IκB kinases (IKKα and IKKβ) phosphorylate IkBα to promote its degradation after its ubiquitination. Once free from IkBα, the active heterodimer p50/p65 is imported into the nucleus prior to recognition of its binding sites along the host cellular genome and the HIV-1 5′LTR. Then, the heterodimer complex is able to recruit histone acetyltransferases (HATs) to promote histone acetylation and therefore HIV-1 transcription. In addition, a recent study has shown that the RelB effector, a member of the NF-κB family, interacts with the viral transactivator Tat to promote its recruitment to the HIV-1 promoter and transactivate viral transcription, therefore demonstrating that NF-κB positively modulates HIV-1 gene expression through various mechanisms in both basal and transactivated conditions [16].

However, even if NF-κB binding to the 5′LTR enhancer region is able to activate HIV-1 transcription, the co-recruitment of additional cellular factors allows a more efficient transcriptional activation. Among these additional factors, Sp1 and AP-1 have been shown to synergically promote viral gene expression through protein–protein interactions [14,17], in collaboration with NF-κB. Furthermore, some host cellular factors share the same binding site and can thus compete for NF-κB binding. Indeed, while NFAT, a member of the Rel transcription factor family, is involved in HIV-1 transcription activation by being recruited to the κB sites in the enhancer region [18,19], HspBP1 (HSP70 binding protein 1) has been reported as an endogenous negative regulator of HIV-1 gene expression by repressing the NF-κB-mediated activation [20]. Moreover, HIV-1 transcriptional initiation can also be repressed by the NF-κB homodimer p50/p50 through HDAC recruitment, therefore promoting viral latency [21].

Among the “shock and kill” approaches, one of the most studied strategy is the use of PKC agonists, such as bryostatin and prostratin, in order to promote HIV-1 gene expression in the latent reservoirs through activation of the NF-κB pathway [22]. Nevertheless, global NF-κB activators are not specific to HIV-1 activation and can exhibit cellular toxicity, preventing their use as therapeutic drugs. Interestingly, it has been shown that HIV-1 latent reservoirs mostly express IκBε isoform instead of the classical isoform IκBα with, as consequences, a prevention of NF-κB-mediated transcriptional activation. Functionally, the specific knocked-down of IκBε promotes HIV-1 expression by increasing the NF-κB binding to the 5′LTR, therefore suggesting that targeting specifically IkBε could be a more specific manner to reverse HIV-1 latency [23].

#### 2.1.2. Sp1

Among the key cellular transcriptional factors required to initiate HIV-1 transcription, Sp1 physically interacts with the 5′LTR core promoter via three G-C rich *cis*-regulatory binding sites, located just downstream of the NF-κB binding sites [10]. After cellular stimulation, Sp1 acts synergistically with NF-κB via protein–protein interactions to activate viral transcription [14]. Indeed, it has been shown that Sp1 and NF-κB both interact with TBP and some TBP associated-factors (TAFs), such as TAF_II_250 for NF-κB (through the p65 subunit) and TAF110 for Sp1, which are components of the general RNAPII transcriptional factor D (TFIID) [24,25]. In summary, NF-κB and Sp1 are key transcription factors to recruit the RNAPII-dependent transcriptional machinery to the HIV-1 promoter. In addition, this interaction has been shown to induce a chromatin reconfiguration promoting transcriptional activation [26,27], mediated by the interaction between Sp1 and both HDACs and HATs [28,29]. In HIV-infected microglial cells, which constitute the major HIV-1 reservoir in the brain, Sp1 has been shown to physically interact with the nuclear factor chicken ovalbumin upstream promoter transcription factor (COUP-TF) to synergically activate viral transcription from the 5′LTR [30]. In this study, the authors showed that under latent conditions, the COUP-TF interacting protein 2 (CTIP2) represses Sp1- and COUP-TF-mediated activation of HIV-1 transcription through the establishment of a repressive epigenetic environment, by the recruitment of HDACs (HDAC1 and HDAC2), of the histone methyltransferase (HMT) Suv39H1, catalysing trimethylation of lysine 9 of histone H3 (H3K9me3), and of HP1 (heterochromatin protein 1) [31,32].

In addition, TRIM22, a member of the TRIpartite motif (TRIM) family which is strongly induced upon interferon stimulation, has recently been described as a new potential therapeutic target. Indeed, it has been shown that TRIM22 represses the Tat- and NF-κB-mediated viral transcriptional activity independently of its E3 ligase activity, by interfering with Sp1 binding at the HIV-1 core promoter and therefore preventing formation of the transcriptional pre-initiation complex. TRIM22 is therefore a target candidate in order to reverse viral latency in HIV-1 reservoirs [33,34,35]. In the context of the “block and lock” strategies, the interferon γ-inducible protein 16 (IFI16) has been described as a putative new therapeutic agent to block viral transcription by sequestrating Sp1, thereby preventing the Sp1-mediated transcriptional activation of the HIV-1 5′LTR [36].

#### 2.1.3. The CATA Box and the Initiator Element

To recruit RNAPII, the 5′LTR core promoter also contains a non-canonical TATA box, called the CATA box (CATATAA), which is an important DNA sequence used by 12% of cellular RNAPII-dependent promoters, allowing a basal promoter activity [37]. Indeed, this TATA box variant is bound by the TATA Binding Protein (TBP), a subunit of the general RNAPII transcription factor TFIID, to induce the formation of the transcription pre-initiation complex. However, this CATA motif is not the same for all the HIV-1 subtypes. Indeed, it has been shown that the subtype E contains a single nucleotide polymorphism T/A in the CATA motif (CATA**T**AA to CATA**A**AA), which still allows the RNAPII-dependent transcriptional activity of the 5′LTR. However, in vitro experiments have shown that this subtype E exhibits a lower viral replication fitness compared to the other HIV-1 subtypes containing the canonical CATA motif (CATA**T**AA). Moreover, a point mutation in the CATA box (**C**ATATAA mutated in **T**ATATAA) is associated with an increase in HIV-1 replication, suggesting that mutation hampering HIV-1 promoter activity has been selected for the viral fitness [37].

Downstream of the CATA box, the HIV-1 core promoter contains an initiator (Inr) element to promote transcriptional initiation [10]. This Inr contains a highly conserved *cis*-regulatory element called RBEI, known to bind the RBF-2 complex (TFII-I and USF1/2). Interestingly, three other RBE elements were found upstream of the HIV-1 core promoter. RBEII and RBEIV have been described to be bound by the RBF-1 complex, while RBEIII is bound by the RBF-2 complex similarly to RBEI. Overall, the role of the RBF-2 complex in HIV-1 transcription has been the most studied. In unstimulated cells, TFII-I may function as a transcriptional repressor by recruiting the histone deacetylase 3 (HDAC3), thereby promoting histone deacetylation. The engagement of T cell receptor (CD3-CD4) induces the activation of the Ras/MAPK signalling pathway through the production of the second messenger’s diacylglycerol (DAG). Then, phosphorylation of all the subunits of the RBF-2 complex induces the recruitment of HATs, thereby promoting histone acetylation and HIV-1 transcription [38,39]. Moreover, the functional effect of the RBF-2 complex may also be dependent on the recruitment of the ying-yang factor 1 (YY1) to the HIV-1 promoter. YY1 has been reported to act as a transcriptional repressor of the 5′LTR promoter activity through the recruitment of HDAC1 [40,41]. Interestingly, the two YY1 binding sites found in the 5′LTR are located near the RBF-2 binding sites (RBEI and RBEIII). In terms of binding, YY1 is known to be indirectly recruited by the late simian virus 40 factor (LSF) at the initiator element (−17, +27), close to RBEI, while YY1 is directly recruited at the modulatory region (−140, −120), encompassing RBEIII. Currently, it has not been reported if the recruitment of RBF-2 and YY1 to the RBEIII site is mutually exclusive or simultaneous. However, it has been reported that a PMA (phorbol 12-myristate 13-acetate) stimulation of HIV-1 latently infected T cells induces a decrease in YY1 recruitment at the 5′LTR, while RBF-2 seems to be constitutively recruited [40]. 

### 2.2. The cis-Regulatory Elements Outside the 5′LTR

In addition to the *cis*-regulatory elements found in the 5′LTR, the characterization of the chromatin organization of the entire HIV-1 provirus and the subsequent nucleosomal positioning have revealed two others *cis*-regulatory regions that regulate viral transcription. Indeed, by performing indirect end-labelling experiments on HIV-1 chronically infected monocytes and T-lymphocytes, it has been shown that the HIV-1 provirus harbours a specific nucleosomal chromatin organization. Under latent conditions, two major DNaseI hypersensitive sites (HS), named HSII (from nt −234 to nt −132) and HSIII (from nt −67 to nt −7), have been found in the 5′LTR (Figure 1C). These HS correspond to “nucleosome-free” DNA sequences that are therefore more accessible to transcription factors [42]. In addition to the HS identified in the 5′LTR, two other major HSs, called HSIV and HSVII, have been identified outside the 5′LTR. While the HSIV is located upstream of the leader region of the 5′LTR in both cellular contexts of HIV-1 infection (T-lymphocytes and monocytes), the HSVII is located at the end of the *pol* gene and appears only in HIV-1 chronically infected monocytic cells, suggesting a cellular specificity of this HSVII in the HIV-1 transcriptional regulation of infected monocytes. Overall, these two major HS correspond to *cis*-regulatory regions, which encompass multiple binding sites for transcriptional activators and repressors in order to modulate the basal HIV-1 promoter activity [42]. Concerning the HSIV region, located immediately downstream of the transcription start site (TSS), several studies have identified *cis*-regulatory elements for several transcription factors including AP-1, IRF, Sp1, ATF, CREB, and NFAT [11,43]. By using viruses mutated in these transcription factor binding sites, it has been shown that they play a crucial role in both HIV-1 replication and transcription [43]. Interestingly, the HSVII is a part of a functional region called the intragenic *cis*-regulatory region (IRR), which encompasses the *pol*, *vif*, *vpr*, and a part of the *tat* viral genes [44]. This region is composed of three functional domains: the fragment 5103 (from nt +4079 to nt +4342) and the fragment 5105 (from nt +4781 to nt +6026), which exhibit both a PMA-inducible enhancer activity on a heterologous promoter and flank the third domain corresponding to the HSVII (from nt +4481 to nt +4982) [42,44,45]. Over the years, our laboratory has identified various binding sites for cellular transcription factors in the IRR, such as Oct-1, AP-1, Est-1, Sp1, and PU.1 [45,46]. Furthermore, we have reported that the combined mutation in three AP-1 binding sites along the IRR induces a decrease in RNAPII recruitment to the 5′LTR in vivo, which is associated with a lower viral replication rate, demonstrating that the three AP-1 binding sites in the IRR are responsible for the PMA-inducible enhancer activity of the intragenic region on the HIV-1 5′LTR promoter [47].

### 2.3. The Nucleosomal Organization of the 5′LTR

As described above, the functional effects of transcription factors on HIV-1 promoter activity depends on the recruitment of co-activators (HATs) and co-repressors (HDACs), which induce epigenetic changes by post-translationally modifying histone tails, leading to chromatin remodelling. Such epigenetic modifications are key elements allowing the fine-tuned regulation of HIV-1 transcription. Indeed, as cellular genes, once integrated into the host genome, the provirus adopts a specific chromatin organization, contributing to the regulation of HIV-1 transcription through the specific positioning of nucleosomes. By performing indirect-end labelling assays using micrococcal nuclease (MNase) digestion, the nucleosomal organization of the HIV-1 provirus along the 5′LTR has been depicted. Interestingly, this nucleosomal organization is strictly defined and is independent of the integration site. The HIV-1 5′LTR contains two nucleosomes, named nuc-0 and nuc-1, which flank the nuclease hypersensitive sites HSII and HSIII [48]. Under latent conditions, in absence of the viral transactivator Tat, nuc-1 is actively positioned just downstream of TSS and acts as a powerful repressor by interfering with the transcriptional elongation process. Indeed, the BRG1- or HBRM-associated factor (BAF) complex, an ATP-dependent chromatin remodeler belonging to the SWI/SNF family, actively maintains nuc-1 positioning in order to repress HIV-1 transcription [49].

**Figure 2:**
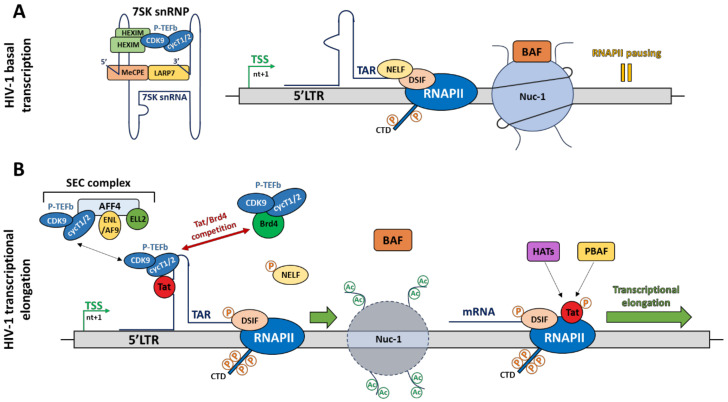
Schematic representation of the molecular mechanisms involved in the regulation of HIV-1 transcription. HIV-1 transcription is regulated at two steps: the basal transcription and the transcriptional elongation. (**A**) The initiation of HIV-1 transcription is strongly regulated through *cis*-regulatory elements located in the 5′LTR. The subsequent recruitment of cellular transcription factors to their binding sites is sufficient to promote the recruitment of the transcription pre-initiation complex and thus to initiate HIV-1 transcription. However, the transcriptional elongation is paused due to (1) the recruitment of two pausing factors, called DRB (5,6-dichloro-1-β-d-ribofuranosylbenzimidazole) sensitivity-inducing factor (DSIF) and negative elongation factor (NELF), on the RNA polymerase II (RNAPII) and to (2) the positioning of nuc-1, mediated by BRG1- or HBRM-associated factor (BAF), just downstream of the TSS. HIV-1 transcription is then aborted and results in the formation of the trans-activation response (TAR) element. During this step, the positive transcriptional elongation factor b (P-TEFb) is sequestered under an inactive form in the 7SK snRNP complex. (**B**) The transcriptional elongation is mainly regulated by the viral transactivator Tat. Tat is recruited at the TAR element and then recruited itself P-TEFb, thereby inducing the phosphorylation of the RNAPII carboxy terminal domain (CTD) and of the pausing factors. Furthermore, nuc-1 is remodelled upstream of the TSS, promoting an efficient transcriptional elongation. The super elongation complex (SEC) is also involved in the recruitment of P-TEFb. During the transcriptional elongation process, P-TEFb and Tat undergo multiple association/dissociation cycles allowing a powerful induction of HIV-1 gene expression.

Mechanistically, the BAF complex, containing the specific subunit BAF250a, is recruited to position nuc-1 downstream of the TSS through a process involving ATP hydrolysis and thus, generates a repressive chromatin conformation by pausing the transcriptional elongation (Figure 2A). However, in activated conditions (i.e., in presence of Tat), the BAF complex is released with, as consequences, a repositioning of nuc-1 upstream of the TSS. Then, the polybromo-associated BAF (PBAF) complex is recruited by the acetylated viral protein Tat to promote a positive chromatin remodelling, enabling an efficient transcriptional elongation [49] (Figure 2B). Currently, the process allowing BAF recruitment to the HIV-1 promoter is unknown. However, the transcriptional repressor YY1, known to be recruited under latent conditions at the initiator element by LSF [41,49], could be a putative candidate. In addition to the BAF-induced chromatin remodelling mechanism, a Tat-independent mechanism has also been described. More specifically, the specific depletion of the SWI/SNF complex containing BRM as catalytic subunit, induces the repression of Tat-independent transcriptional elongation [50]. Recently, it has been shown that the short isoform BRD4S, a member of the BET family, acts as a corepressor of HIV-1 transcription by a direct binding to the catalytic subunit BRG1 of the BAF complex, thereby promoting nuc-1 remodelling downstream of the TSS [51]. Furthermore, the use of JQ1, a BET inhibitor, promotes the release of BRD4S from BRDG1 and induces the dissociation of the BAF complex from the HIV-1 promoter [51]. The use of BET inhibitors might thus offer interesting perspectives regarding the “shock and kill” strategy to cure HIV-1 infection. In this regard, a synergistic activation of HIV-1 promoter activity has been observed by combining BET inhibitors with the PKC agonist prostratin [52]. Currently, several macrolactam molecules have also been shown to induce HIV-1 transcription from infected CD4+ T cells by preventing BAF recruitment to the 5′LTR through its interaction with the subunit ARID1A. The combination of different classes of molecules targeting the BAF complex could thus allow us to reach interesting levels of HIV-1 transcriptional reactivation [52].

### 2.4. The Pre-Initiation Complex Formation

Initiation of HIV-1 transcription is strongly regulated through *cis*-regulatory elements located in the 5′LTR and in the IRR. The subsequent recruitment of cellular transcription factors to their binding site is sufficient to promote the recruitment of the transcription pre-initiation complex and thus to initiate HIV-1 transcription. The first step in the assembly of the transcription pre-initiation complex is the binding of TBP, a TFIID subunit, to the CATA box and is regulated by the interaction between transcription factors (such as Sp1 and NF-κB) and other TAFs subunits of TFIID. Then, the other RNAPII-dependent general transcriptional factors (TFIIA, TFIIB, TFIIF, TFIIE, and TFIIH) and the 25 subunits of the coactivator complex mediator are recruited prior to the RNAPII recruitment (reviewed in [53,54]). Among those factors, TFIIH is required to initiate transcription through its helicase activity, allowing the transcription of a single-stranded DNA. The phosphorylation state of the RNAPII carboxy-terminal domain (CTD), composed of 52 repeats of a seven amino acids sequence (Tyr-Ser-Pro-Thr-Ser-Pro-Ser), determines its processivity. Indeed, once the transcription pre-initiation complex is recruited to the HIV-1 promoter, the CTD is phosphorylated on serine 5 (Ser5) residues by the cyclin-dependent kinase 7 (Cdk7), a subunit of TFIIH. This modification favours transcription initiation by inducing the release of the RNAPII from the promoter [53,55]. However, in the absence of the HIV-1 transactivator Tat, the transcriptional elongation is not efficient and is rapidly aborted after stalling of the elongation complex approximatively 60 nucleotides downstream of the TSS. This short elongation process leads to the transcription of the leader region of the HIV-1 5′LTR and generates a short nascent RNA transcript of 59 nucleotides called the trans-activation response element (TAR). The TAR RNA plays a critical role in mediating efficient HIV-1 transcription following viral activation (see Section 3.1).

### 2.5. RNA Polymerase II Pausing

Promoter-proximal pausing is not specific to HIV-1 transcription, since it has been described for many eukaryotic promoters that are subject to a fine-tuned regulation of their gene expression rate [56]. RNAPII pausing, corresponding to the second step allowing an efficient transcription, is due to the binding of two pausing factors, the negative elongation factor (NELF) and the DRB (5,6-dichloro-1-β-d-ribofuranosylbenzimidazole) sensitivity-inducing factor (DSIF), as well as to a closed chromatin conformation through the positioning of nuc-1 [49,57,58] (Figure 2A). It is well established that NELF pauses the transcriptional elongation only in the presence of DSIF and that both factors act together to block RNAPII processivity. Mechanistically, DSIF first binds the nascent RNA through its subunit Spt5 prior to the recruitment of NELF [59,60]. Functional studies have demonstrated that NELF depletion in infected primary T cells increases HIV-1 transcription, thereby suggesting that this pausing factor plays a critical role in RNAPII pausing associated with HIV-1 latency. Indeed, NELF is known to interact with the pre-mRNA-cleavage complex II factor (Pcf11), inducing the promoter-proximal pausing and the pre-mature termination through the RNAPII dissociation from DNA, as well as with the repressor complex composed of the nuclear corepressor (NCoR1), the G protein pathway suppressor 2 (Gps2) and the histone deacetylase 3 (HDAC3), promoting a negative epigenetic landscape along the provirus to prevent HIV transcription [57,61,62]. Finally, NELF recruitment could also be enhanced following its interaction with an NBE-like element of TAR via its NELF-E subunit (containing an RNA binding domain) [63].

## 3. Regulation of HIV-1 Transcriptional Elongation

### 3.1. Tat-Mediated Transcription

Among the multiple steps of HIV-1 transcriptional regulation, the transcriptional elongation step is the most regulated. This process is mainly regulated by the human positive transcriptional elongation factor b (P-TEFb), which interacts with the critical protein-RNA complex composed of the viral transactivator Tat and the nascent HIV-1 RNA TAR (Figure 2B). The HIV-1 Tat protein is the strongest HIV-1 activator and promotes the RNAPII escape from the HIV-1 promoter to allow an efficient transcriptional elongation. However, this Tat-mediated HIV-1 transcriptional elongation is a multistep mechanism. First, Tat binds TAR through its basic ARM (arginine-rich RNA-binding motif) domain at the UCU bulge of TAR near its major loop. It has been shown that the arginine 52 (R52) residue within the ARM domain of Tat is crucial for Tat-TAR interaction and is therefore essential for viral transactivation [64,65]. Upon stimulation, the cellular factor P300/CBP-associated factor (PCAF) acetylates Tat on its lysine 28 (K28) residue to mediate P-TEFb recruitment to the Tat-TAR complex [66]. P-TEFb is an heterodimer composed of the cyclin T1 or T2, interacting with K28-acetylated Tat, and the cyclin-dependent kinase 9 (CDK9), which phosphorylates the RNAPII CTD on its serine 2 residue to switch the poised RNAPII to the elongated one [67]. Furthermore, CDK9 also phosphorylates DSIF on its Spt5 subunit, thereby converting this pausing factor into a positive elongating factor, as well as the NELF-E/DR subunit of NELF to induce its release from the HIV-1 promoter and to remove the transcriptional elongation block [58,68,69]. During the elongation process, Tat is acetylated on its lysine 50 (K50) residue by the cellular proteins hGCN5 and/or p300 to release the Tat-P-TEFb complex from TAR and promote its subsequent transfer to the RNAPII-dependent transcriptional elongation complex [70,71]. However, it has been shown that P-TEFb and Tat undergo multiple association/dissociation cycles during HIV-1 transcriptional elongation, allowing a powerful induction of HIV-1 gene expression [72]. In parallel to this mode of action, Tat also recruits HAT (such as PCAF) and the chromatin remodeler PBAF to promote euchromatin formation and to favour the transcriptional elongation step [49,71] (Figure 2B). At the end of this process, Tat is deacetylated by the sirtuin 1 (SIRT1), a member of class III HDAC family, allowing its release from the RNAPII. Then, the “free” Tat protein can be recycled and recruited again to TAR to start a new cycle of HIV-1 transcription [8,73]. Nevertheless, during HIV-1 latency, this mechanism of transcriptional elongation is blocked due to the unavailability of active P-TEFb complex. Indeed, in latently infected cells, P-TEFb is mostly found in an inactive form due to its interaction with the inhibitory 7SK small nuclear ribonucleoprotein (7SK snRNP) complex (Figure 2A), thereby preventing efficient HIV-1 transcription elongation. Hereafter, we reviewed the P-TEFb-mediated inhibitory mechanisms and how to target them to reactivate HIV-1 in latently infected cells.

### 3.2. The 7SK snRNP Complex

As explained above, the availability of active P-TEFb is a limiting factor to promote Tat-mediated HIV-1 transcription. This factor is usually inactivated through its interaction with the 7SK RNP, a complex composed of the small non-coding RNA 7SK (7SK snRNA), a homo- or heterodimer of the CDK9-inhibitory protein hexamethylene bisacetamide (HMBA)-inducible 1 or 2 (HEXIM 1/2), the methylphosphatase capping enzyme (MePCE), and the La ribonucleoprotein domain family member 7 (LARP7) [74,75,76,77,78,79,80,81] (Figure 2A). The 7SK RNP is thus a negative transcriptional regulatory complex, which inhibits the kinase activity of the elongation factor P-TEFb through its interaction with HEXIM1 or HEXIM2. Functionally, this interaction hides the CDK9 catalytic site [82], thereby preventing transcriptional elongation [75,76,77]. The 7SK snRNA acts as a scaffold platform and is stabilized by the binding of MePCE and LARP7 to its 5′ and 3′ ends, respectively, preventing its cleavage by cellular exonucleases. MePCE and LARP7 can also interact with each other to increase the stability of the 7SK snRNP complex [79,80,81,83]. While the majority of the 7SK snRNP complex is located in nucleoplasmic regions, a small fraction is also found anchored at the proximal-promoter region, associated with the paused RNAPII [84,85]. In the HIV-1 field, an interesting study has reported that the inactive P-TEFb/7SK snRNP complex is recruited to the HIV-1 promoter prior to transcriptional initiation and the production of TAR [84]. An advantage of this 7SK snRNP/P-TEFb accumulation at the HIV-1 promoter is to mediate a fast viral activation upon stimulation by the direct availability of P-TEFb. In addition, several cellular host factors have been shown to be involved in the P-TEFb inactive complex recruitment to the HIV-1 promoter proximal region (reviewed in [86,87,88,89]). 

### 3.3. Mechanisms of 7SK snRNP/P-TEFb Complex Recruitment to Chromatin

#### 3.3.1. KAP1

The Krüppel-associated box (KRAB)-interacting protein 1 (KAP1), also referred to TRIM28 or TIF1β, has been shown to mediate 7SK snRNP complex recruitment by directly interacting with the LARP7 subunit [90]. Interestingly, KAP1 and the 7SK snRNP complex are simultaneously recruited at the HIV-1 promoter-proximal region in CD4+ T-lymphocytic cell lines. This mechanism of recruitment was only observed at the viral promoter containing a transcriptional pre-initiation complex, composed of TBP and Sp1, with a transcriptionally engaged but paused RNAPII [90].

#### 3.3.2. HMGA1 and CTIP2

Another mechanism of 7SK snRNP/P-TEFb inactive complex recruitment to the HIV-1 promoter has been specifically demonstrated in microglial cells, the major source of HIV-1 persistence in the central nervous system. This mechanism involves a cooperation between the non-histone chromatin protein high mobility group AT-hook 1 (HMGA1) and CTIP2 to synergistically repress HIV-1 transcription and promote viral latency [91,92]. CTIP2 has been demonstrated to be a member of the 7SK-snRNP/P-TEFb complex in microglial cells and to repress the CDK9 subunit of P-TEFb, thereby preventing transcriptional elongation [93]. Mechanistically, HMGA1 recruits the CTIP2-containing 7SK snRNP/P-TEFb inactive complex to the viral promoter through its common binding domain with CTIP2 at the stem-loop 2 of the 7SK snRNP [91,92]. Furthermore, CTIP2 also recruits chromatin-modifying enzymes such as HDAC 1/2 and the histone demethylase LSD1 in a Sp1-dependent manner to repress viral transcription [92,94]. This other mechanism of CTIP2-mediated transcriptional repression could also explain the synergistic repression of HMGA1 and CTIP2 on HIV-1 gene expression, although these two mechanisms are likely mutually exclusive [92].

#### 3.3.3. Tat and ZASC1

The 7SK snRNP/P-TEFb inactive complex is also recruited to the HIV-1 promoter in a Tat-dependent manner by the cellular factor ZASC1 also called zinc finger protein 639 (ZNF639) [95]. Indeed, the HIV-1 core promoter contains a conserved *cis*-binding element recognized by ZASC1 and located just upstream of TAR. Mechanistically, ZASC1 is able to directly recruit Tat, the active P-TEFb as well as the 7SK snRNP/P-TEFb inactive complex to the HIV-1 core promoter in a TAR-independent manner [95], thereby promoting HIV-1 transcriptional elongation in T lymphocytes. In addition, Sp1 may also be required for the co-recruitment of Tat/7SK snRNP/P-TEFb inactive complex but has been shown to be insufficient [95].

### 3.4. Release of P-TEFb from the Inhibitory 7SK snRNP Complex

#### 3.4.1. Competition between Tat and HEXIM1

It has been shown that the first 18 amino acids within the NLS domain of HEXIM1 are both sufficient and necessary to bind the 7SK snRNP complex, an essential step to mediate the HEXIM1-dependent inhibition of the P-TEFb kinase activity. Interestingly, this region contains multiple clusters of positively charged amino acids, a homologous feature to the arginine-rich RNA-binding motif found in many proteins. The TAR RNA-binding motif of the HIV-1 protein Tat is highly homologous to this domain but despite similar RNA binding domains, Tat displays an additional arginine residue, called R52, involved in the conformational remodelling of TAR and of the 7SKsnRNA [64,96]. Indeed, it has been previously demonstrated that the interaction between Tat and the 7SKsnRNA induces the release of HEXIM-1 from the 7SKsnRNA and disrupts the inhibition of the kinase activity of P-TEFb. Then, after its binding to TAR, Tat recruits free P-TEFb from the 7SK snRNP complex to promote HIV-1 transcriptional elongation. Moreover, in addition to increasing the pool of active P-TEFb to allow HIV-1 transcription, this interaction between Tat and the 7SKsnRNA also prevents reassembly of the 7SKsnRNP complex [64,97,98] and the subsequent P-TEFb inactivation. Recently, a chimeric inhibitor has been developed to inhibit HIV-1 transcription in the context of the block and lock strategy. This inhibitor, called HT1, consists of the RNA binding domain and the CDK9-inhibitory domain from HEXIM-1 and the P-TEFb binding domain from Tat. Interestingly, HT1 has been demonstrated to effectively block HIV-1 reactivation in latently infected T lymphocytes by competing with the binding of Tat and active P-TEFb to TAR [99].

#### 3.4.2. Competition between Tat and Brd4

Active P-TEFb can also be released from the endogenous cellular protein Brd4. Indeed, while the half of P-TEFb is sequestered in the inactive 7SKsnRNP complex, the remaining half is associated with the bromodomain protein Brd4. Upon stimulation, the inactive form of P-TEFb is converted into its active form, associated with Brd4, to mediate a positive elongation during the transcription of cellular genes [100]. Indeed, Brd4 can directly recruit P-TEFb through its C-terminal domain and interacts with the mediator complex [100]. Furthermore, Brd4 has also been shown to directly displace P-TEFb from the 7SKsnRNP complex through its C-terminal domain [101]. However, Brd4 overexpression experiments have shown a decrease in the interaction between Tat and P-TEFb, thereby interfering with Tat-mediated HIV-1 transactivation [102]. Thus, by comparison to its positive functions on the transcriptional elongation of cellular genes, Brd4 has a negative role on the regulation of HIV-1 transcription by competing with Tat for the binding to P-TEFb. Thus, the inhibition of Brd4 has been proposed in the context of the “shock and kill” strategy. Indeed, bromodomain and extra-terminal (BET) inhibitors, such as JQ1, have been shown to induce reactivation of HIV-1 transcription by antagonizing Brd4 inhibition of Tat-mediated transcriptional transactivation [103,104]. However, the latency reversal effects of BET inhibitors may also imply Tat-independent processes through the inhibition of Brd2, a Tat-independent repressor of HIV transcription [105]. Interestingly, the combinatory use of JQ1 and prostratin, a PKC agonist, enhances the ability of these drugs to reactivate HIV-1 in vitro, highlighting the importance of combinatorial therapies [104].

#### 3.4.3. Post-Translational Modifications of the 7SK snRNP Complex

Some post-translational modifications have been identified as playing an important role in the release of P-TEFb from the 7SK snRNP complex. The dissociation of HEXIM-1 from the 7SK snRNP complex is the most regulated step to induce the subsequent release of P-TEFb [101]. Indeed, previous studies have shown that upon simulation, HEXIM-1 is phosphorylated on multiple residues, thereby promoting its dissociation from the 7SK snRNA and the release of active P-TEFb. For instance, a PMA stimulation of T lymphocytes induces the phosphorylation of two tyrosine residues in HEXIM-1, at position 271 and 274, and the release of P-TEFb from the 7SK snRNP complex [106]. Furthermore, a stimulation with the hexamethylene bisacetamide (HMBA), a potent activator of HIV-1 transcription, is known to activate the PI3K/Akt pathway, which leads to the phosphorylation of HEXIM-1 on T270 and S278 residues and to the decrease in its interaction with the CycT1 subunit of P-TEFb [107]. 

In addition to the phosphorylation, other post-translational modifications such as pseudouridylation and ubiquitination have been demonstrated to regulate HEXIM-1/P-TEFb interaction. Indeed, the majority of 7SK snRNAs are pseudouridylated on the residue U250 by the DKC1-box H/ACA RNP, a cellular pseudouridine synthetase complex. This modification appears to be crucial for the stability of the 7SK snRNP complex, and it has been shown that either the mutation of residue U250 or the DKC1 depletion induces a disruption of the 7SK snRNP complex, thereby activating HIV-1 transcription through the release of P-TEFb [108]. Another post-translational modification of HEXIM-1 has been demonstrated to play a major role in the release of P-TEFb from the 7SK snRNP complex and in HIV-1 transcriptional regulation. Indeed, the viral transactivator Tat recruits the ubiquitin ligase UBE2O to ubiquitinate HEXIM-1 in a non-degradative manner in order to induce the release of active P-TEFb from the 7SK snRNP complex and to induce HIV-1 transcriptional elongation [109]. Interestingly, this UBE2O-dependent ubiquitination of HEXIM-1 is mainly cytoplasmic, leading to the discovery of a cytoplasmic pool of P-TEFb/7SK snRNP complex in addition to the nucleoplasmic one. UBE2O plays therefore a crucial role in the release of active P-TEFb from its cytoplasmic pool before its nuclear import and its Tat-dependent recruitment to the HIV-1 promoter [109].

Post-translational modifications of P-TEFb have also been described to regulate its release from the 7SK snRNP complex. For instance, the HIV-1 transactivator Tat has been shown to recruit to the viral promoter the phosphatase PPM1G/PP2Cꙋ to locally activate P-TEFb. Mechanistically, PPM1G dephosphorylates the T-loop of CDK9 on the residue Thr186, inducing the release of P-TEFb from the 7SK snRNP complex. Interestingly, the NF-KB factors have also been shown to recruit PPM1G in order to activate inflammatory gene response upon stimulation [85,110]. Once P-TEFb is released, PPM1G directly binds to the 7SK snRNA and HEXIM-1 to block P-TEFb reassembly in the 7SK RNP complex [110].

### 3.5. The Super Elongation Complex (SEC)

Affinity-purification experiments have revealed that the Tat/P-TEFb complex is interacting with many proteins members of the super elongation complex (SEC). This multiproteic complex is essential to promote an efficient transcriptional elongation and includes a scaffold protein (AFF1/4), co-factors (ENL and AF9) and the positive elongation factor ELL2 [111,112] (Figure 2B). The SEC is recruited by the viral protein Tat, which binds to the scaffold protein AFF4. This binding increases the affinity between AFF4 and the cyclin T subunit of P-TEFb, thereby allowing their physical interaction [113]. The viral protein Tat also promotes the formation of the SEC by increasing the stability of the elongation factor ELL2 in a CDK9-dependent manner. Indeed, Tat has been shown to increase the sequestration of ELL2 in the SEC to promote a SEC-dependent efficient elongation [111,112]. Once recruited, the positive elongation factor ELL2 acts cooperatively with P-TEFb to promote an efficient Tat-mediated elongation. In addition, ELL2 is known to stimulate the transcriptional elongation by suppressing the transient pausing of RNAPII [114,115]. However, the SEC can also act in a Tat-independent manner. The scaffold protein AFF4 has been shown to mediate the interaction between SEC and P-TEFb and to play a role similar to the one of Tat by increasing the stability of ELL2 and inducing SEC assembly. Thus, overexpression of ELL2 and AFF4 could be a new therapeutic strategy to reverse HIV-1 latency in absence of Tat [111,112].

The availability of ELL2 is a limiting factor for SEC assembly. This elongating factor has a short lifetime and is rapidly degraded by the proteasomal pathway. To address ELL2 to the proteasome, the E3 ubiquitin ligase Siah1 plays a crucial role by promoting the degradation of ELL2 through its ubiquitination. At high concentrations, Siah1 also degrades AFF1/4 to prevent the formation of the SEC [116]. Thus, in line with the shock and kill strategy, targeting Siah1 is a putative new strategy to promote HIV-1 gene expression. In agreement, the combinatory use of prostratin and HMBA, two HIV-1 transcriptional activators, promotes ELL2 accumulation, favouring the SEC assembly, by decreasing Siah1 expression [116]. 

The SEC-mediated transcriptional elongation process is also regulated by a specific CTD phosphatase called Ssu72. This enzyme mediates the phosphorylation of the RNAPII CTD on its residues Ser5 and Ser7, without affecting the phosphorylation of Ser2, thereby promoting the transition from paused RNAPII to elongated one [117,118]. Interestingly, Ssu72 is recruited by the viral protein Tat simultaneously with P-TEFb and the SEC complex to drastically induce transcriptional elongation [119]. Because Ssu72 is not an essential factor required for cellular gene transcription, it could be a new drug target in order to block HIV-1 gene expression, a major aim of the block and lock strategy [117,119].

The epigenetic profile along the HIV-1 5′LTR in latently infected cells is also important for the recruitment of the SEC. Indeed, during HIV-1 latency, the 5′LTR is characterized by a high acetylation of histone 4 (AcH4), favouring the recruitment of Brd4 to the promoter and preventing the loading of the SEC to Tat. Interestingly, the depletion of the lysine acetyltransferase KAT5, responsible for H4 acetylation, is associated with a decrease in Brd4 recruitment and an increase in SEC recruitment to Tat in order to favour HIV-1 transcription [120].

In addition of the scaffold protein AFF4, the SEC complex can also contain another scaffold protein of the AF4/FMR2 family, called AFF1, and play another role in HIV-1 transcriptional regulation. Indeed, while the SEC-containing AFF1 has been shown to be important for Tat-mediated transactivation, the SEC-containing AFF4 activates HIV-1 transcription in a Tat-independent manner [121]. In agreement with this observation, the double-depletion of AFF4 and AFF1 abolishes both Tat-mediated and Tat-independent HIV-1 transcription [121]. In addition, AFF4 and to a lesser extent AFF1 are also recruited to the HIV promoter through their association with the 7SK snRNA as well as with TAR RNA [121]. Taken together, the SEC-containing AFF4 seems to promote the early steps of HIV-1 transcription (in the absence of Tat), while the SEC-containing AFF1 is preferentially used to enhance Tat-mediated transactivation of the HIV-1 promoter.

## 4. Conclusions

Over the past 30 years, the introduction of cART has drastically reduced AIDS-related mortality and saved millions of people lives worldwide. However, the establishment of HIV-1 latency remains the major barrier to reach an HIV-1 functional cure. To overcome this issue, the scientific community has made ground-breaking discoveries allowing us to better understand the broad range of molecular mechanisms regulating HIV-1 transcription. In addition, through those discoveries, new therapeutic approaches aiming at activating viral transcription have emerged to purge HIV-1 latent reservoirs and to finally reach the golden goal of an HIV-1 functional cure. However, even if many transcriptional mechanisms involving various cellular factors have already been extensively studied, our understanding of HIV-1 transcriptional regulation is always evolving and will definitively lead to new discoveries and putative more efficient therapeutic targets. Another major issue is to better explore the implications of all those cellular factors in viral transcriptional regulation in HIV-infected patients in which the transcriptional mechanisms regulating HIV-1 gene expression have been shown to be more complex and heterogeneous. Indeed, it has been demonstrated that the molecular mechanisms underlying HIV transcription and latency vary among patients and are potentially associated with specific clinical features. Therefore, the main future interest in the HIV-1 transcription field is to conduct research in a more physiological context of HIV-1 infection, using HIV-1 infected primary cells, to provide the more adapted therapies for infected patients.
